# Supplementary material for: Topological transformations of a nematic drop
Source: Sci Adv. 2023 Jul 7;9(27):eadf3385. doi: 10.1126/sciadv.adf3385 (PMC10328399; doi:10.1126/sciadv.adf3385)
Supplement: Supplementary file 1 — Figs. S1 to S4 [file sciadv.adf3385_sm.pdf]

Science Advances
AAAS

Supplementary Materials for

Topological transformations of a nematic drop

Runa Koizumi *et al.*

Corresponding author: Dmitry Golovaty, dmitry@uakron.edu; Oleg D. Lavrentovich, olavrent@kent.edu

Sci. Adv. **9**, eadf3385 (2023)
DOI: 10.1126/sciadv.adf3385

This PDF file includes:

Figs. S1 to S4

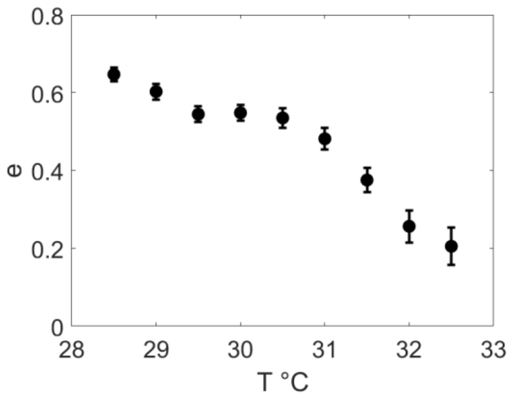

Fig. S1. Temperature dependence of the eccentricity of ellipsoidal N droplets of the aqueous dispersion of DSCG+PEG. $c = 0.34$ mol/kg, $C = 0.012$ mol/kg. The error bars correspond to standard deviations in the measurements of 10 droplets.

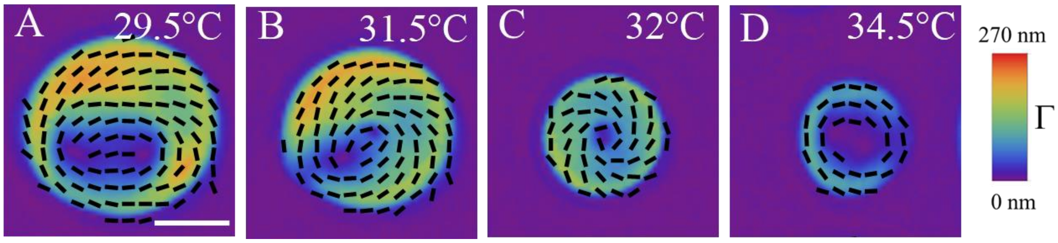

Fig. S2. Temperature-triggered tactoid-to-toroid transformation with two disclinations coalescing away from the center. Aqueous dispersion of DSCG+PEG, $c = 0.34$ mol/kg and $C = 0.012$ mol/kg. Scale bar 10 μm.

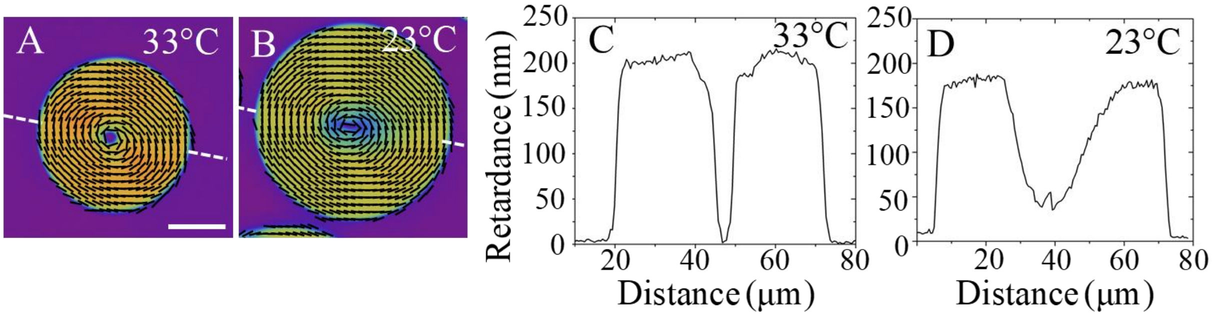

Fig. S3. Nematic droplet shape changes upon cooling. DSCG+PEG dispersion, $c = 0.34$mol/kg , $C = 0.011$mol/kg. (A,B) Polscope textures at $T = 33$°C and 23°C, respectively. Scale bar 20 μm. (C,D) Corresponding profiles of optical retardance measured along the white dashed line in (A,B). In (D), the splitting of the disclination core is visible, as evidenced by a small peak located at ≈ 40 μm.

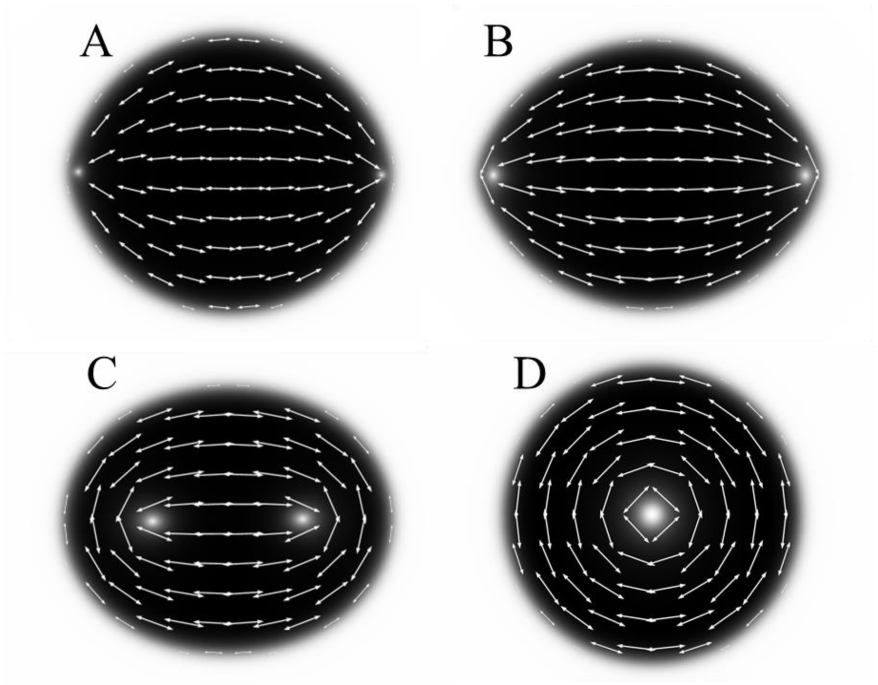

Fig. S4. Numerically simulated equilibrium shapes of N droplets for different values of the splay modulus K_{11}; $\gamma = 10^{-4}$ J/m^2 and $K_{33} = 25$ pN are fixed, while K_{11} increases: (A) $K_{11} = 5$ pN, (B) 24 pN, (C) 80 pN, (D) 120 pN.
